# Authors' reply

**Published:** 2009-10

**Authors:** Kavitha S. Nair, Kirti V. Patel, Tejal R. Gandhi

**Affiliations:** Department of Pharmacology, Anand Pharmacy College, Opp Town Hall, Anand - 388 001, Gujarat, India. E-mail: kavitha21_82@yahoo.co.in

Sir,

This is with reference to the comments on our paper as described above.[1] Few questions have been posed and the answers to the same are as follows:

1. It is true that NADPH is a cofactor for aldose reductase. The electrons are, however, supplied from NADPH via cytochrome P450 reductase, which is closely associated with cytochrome P450 in the lipid membrane of the smooth endoplasmic reticulum.[2] Thus, any reaction that will alter the synthesis of NADPH will be able to affect the aldose reductase pathway. However, in the current context, the synthesis of NADPH is not targeted as hypothesis, whereas the electron transfer from NADPH has to be targeted through cytochrome P450 modulation (i.e. either inhibition or induction).
2. It should be noticed that cytochrome P450 is present at various sites in the body, one of which is lens where the presence of the aldose reductase pathway is not debatable. Using phenobarbital, the occurrence of cataract through cytochrome P450 modulation has been amply demonstrated.
3. The cytochrome P450 induction will in no way affect the concentrations of NADPH but will increase the rate of reaction catalyzed by aldose reductase through supporting the electron transfer from NADPH. This forms the basis of hypothesis, whereby induction of cytochrome P450 is proposed to increase the occurrence of cataract and vice versa.
4. Even a minor amount of unbound drug could have a significant role to play. Moreover, the scope of an activity cannot be limited to just one factor alone.
5. It appears that the cytochrome modulatory role of pioglitazone is doubtful. However, an ample evidence has accumulated to support its role as a cytochrome P450 enzyme inducer (although weak).[3,4]
6. Galactose has not been used for induction of diabetes but for the induction of cataract. This is attributed to accumulation of galactitol in the lens. Rats fed with excess galactose accumulate galactitol within the lens. This results in an osmotic effect bringing in water and causing swelling and opacification. The key enzyme for this chain of action in lens is aldose reductase (AR).
